# Racial/Ethnic Disparities in Prostate Cancer 5-Year Survival: The Role of Health-Care Access and Disease Severity

**DOI:** 10.3390/cancers15174284

**Published:** 2023-08-27

**Authors:** Christiane J. El Khoury, Sean A. P. Clouston

**Affiliations:** 1 Program in Public Health, Renaissance School of Medicine at Stony Brook, Stony Brook, NY 11790, USA; sean.clouston@stonybrookmedicine.edu; 2 Department of Medical Oncology, The Sidney Kimmel Cancer Center at Thomas Jefferson University, Philadelphia, PA 19123, USA; 3 Department of Family, Population and Preventive Medicine, Renaissance School of Medicine at Stony Brook, Stony Brook, NY 11790, USA

**Keywords:** prostate cancer, survival, disparities, geographic, multilevel, healthcare access

## Abstract

**Simple Summary:**

This study explores the five-year survival rates of prostate cancer patients (PCa) in the United States, considering their socioeconomic status (SES) and discussing the role of healthcare access and disease severity. A population-based incidence database was used to examine the five-year survival of PCa patients, considering factors such as county-level SES and healthcare access and utilization. The results showed that living in counties with lower income and education levels was linked to higher PCa mortality rates, while better education levels were associated with lower mortality. However, associations varied depending on race and ethnicity. This study suggests that access to healthcare and the stage/grade appear to influence the relationship between county-level SES and survival rates. Our findings highlight the need for tailored interventions to address these disparities and could guide future research into improving PCa survival rates by considering area-level characteristics and demographic profiles.

**Abstract:**

Introduction: Prostate cancer (PCa) exhibits one of the widest racial and socioeconomic disparities. PCa disparities have also been widely linked to location, as living in more deprived regions was associated with lower healthcare access and worse outcomes. This study aims to examine PCa survival across various USA counties in function of different socioeconomic profiles and discuss the role of potential intermediary factors. Methods: The SEER database linked to county-level SES was utilized. Five-year PCa-specific survival using the Kaplan–Meier method was performed for five racial/ethnic categories in function of SES quintiles. Multilevel Cox proportional hazards regression was performed to assess the relationship between county-level SES and PCa survival. Multivariate regression analysis was performed to examine the role of healthcare utilization and severity. Results: A total of 239,613 PCa records were extracted, and 5-year PCa-specific survival was 94%. Overall, living in counties in the worst poverty/income quintile and the worst high-school level education increased PCa mortality by 38% and 33%, respectively, while the best bachelor’s-level education rates decreased mortality risk by 23%. Associations varied considerably upon racial/ethnic stratification. Multilevel analyses showed varying contributions of individual and area-level factors to survival within minorities. The relationship between SES and PCa survival appeared to be influenced by healthcare utilization and disease stage/grade. Discussion: Racial/ethnic categories responded differently under similar county-level SES and individual-level factors to the point where disparities reversed in Hispanic populations. The inclusion of healthcare utilization and severity factors may provide partial early support for their role as intermediaries. Healthcare access (insurance) might not necessarily be associated with better PCa survival through the performance of biopsy and or/surgery. County-level education plays an important role in PCa decision making as it might elucidate discussions of other non-invasive management options. Conclusions: The findings of this study demonstrate that interventions need to be tailored according to each group’s needs. This potentially informs the focus of public health efforts in terms of planning and prioritization. This study could also direct further research delving into pathways between area-level characteristics with PCa survival.

## 1. Introduction

Prostate cancer (PCa) is the second leading cause of death and the most common cancer in men residing in the USA [1]. Although survival from PCa is relatively very high compared to that associated with other malignancies, remarkable disparities in PCa outcomes have been reported across multiple settings and series. For example, patients reporting Black race/ethnicity have a 78% higher incidence of PCa as compared to White patients in their lifetimes [1,2,3,4]. PCa is more aggressive and occurs at a younger age in Black men, resulting in a 2.3-fold increase in mortality rate as compared to White men [3,4,5]. Hispanic men, and some populations of Asian descent, appear to have a lower overall incidence of PCa but suffer from more advanced disease at diagnosis [4,5,6]. 

PCa outcomes are sensitive to geographic locations [7], as disparities vary based on area-level characteristics at multiple geographic scales [7,8,9]. For example, Hispanic individuals living in Mexico have a lower incidence of PCa than Hispanic individuals living in the Caribbean [10], while Puerto Ricans living in Puerto Rico have a lower incidence than Puerto Ricans living in the USA [11], suggesting that geographical variability might help to explain racial/ethnic disparities in PCa survival. 

The existing literature supports the view that disparities emerge, in part, due to inequalities in access to adequate healthcare that vary across race and socioeconomic status [12,13], with some reports suggesting that Black patients are more likely to experience experienced delayed care and reduced risk of surgery [2,14]. Advances in imaging and procedures, and surgical access are critical in PCa [15] as they appear to interfere with disparities in outcomes. For example, lower socioeconomic status and older age were found to be associated with an underutilization of PCa diagnostic imaging in Black patients [15]. More importantly, multi-parametric Magnetic Resonance Imaging (mpMRI) fusion-guided biopsy, a revolutionary technique in accurately diagnosing and staging PCa, was less likely to be performed in Black patients despite having performed substantially better in Black than White patients [16,17]. Disparities in outcomes in PCa after treatment suggest receipt of lower-quality treatment in general [18]. Additionally, disparities decreased in magnitude in equal-access samples like the Veterans Health Administration, indicating non-clinical causal pathways for PCa disparities [19]. 

To date, disparities in PCa survival have been mostly studied across patients reporting either White or Black race/ethnicity, while inequalities have largely examined results from small geographical scales such as neighborhoods or zip codes [20,21,22]. Some evidence suggests that associations between area-level SES and PCa survival differ on the geographical scale chosen [23,24,25]. The goal of the present study was to describe differences in PCa outcomes across an expanded category of racial/ethnic groups, to examine whether county-level SES helped to explain disparities in PCa survival and understand how potential factors influence any established association between county-level SES and PCa survival. 

## 2. Methods

### 2.1. Data Sources and Study Population

Disparities in five-year PCa survival were estimated according to race/ethnicity and SES. The study cohort was derived from the SEER cancer database. The SEER (NCI) program provides valuable information on various cancer statistics as it is a population-based cohort that covers around one-third of the USA population and contains a larger proportion of foreign-born, thereby facilitating studies of racial and ethnic disparities [26]. 

### 2.2. Outcome Definition

The primary outcome of this study is 5-year PCa survival. Cancer-specific mortality was utilized to ascertain net survival and the probability of surviving PCa in the absence of other causes of death. We included biopsy-confirmed diagnoses among men aged 18 years or older. The analyses focused on diagnoses from 1 January 2007 to 31 December 2011, followed up for at least five years through to 31 December 2016. Participants who had missing information on survival follow-up were excluded (11.4%); however, this did not result in a selection bias as no significant differences in racial composition and SES status were found in the excluded proportion. Disease severity was also described through a later SEER summary stage at diagnosis (distant) or a higher Gleason score (GS = 8–10). Healthcare utilization was examined using the risk of surgical intervention and the presence of an undocumented Gleason score. 

### 2.3. Race/Ethnicity and Risk Factors

The groups included all five races/ethnicities within the SEER registry: Non-Hispanic White (Whites), Non-Hispanic Black (Blacks), Non-Hispanic Asian or Pacific Islander (API), Non-Hispanic American Indian or Alaska Native (AI/AN), and Hispanic race/ethnicity. The individual characteristics selected were factors that are known to be associated with PCa survival such as age, marital status, stage at diagnosis, and GS [27,28]. 

### 2.4. Socioeconomic Status (SES)

Area-based SES was derived from the SEER linkage with the American Community Survey (ACS). County-level 5-year ACS results were collected for each case enclosed in its corresponding county. The 2007–2011 5-year ACS linkage was selected as it would reflect the SES of the selected PCa cohort diagnosed between 2007 and 2011. County-level SES represented income (median household income), material deprivation (percentage of individuals 150% below poverty level, percentage of family below poverty level, unemployment rates), social class levels (percentage of individuals with less than high-school education, percentage of individuals with at least a bachelor-level education) and population composition (percentage of foreign-born individuals, percentage of language isolation). Such variables are often included in health outcome research relating poorer SES status to poorer health outcomes such as higher mortality [29,30]. SES characteristics were classified into five quintiles, ranging from worse to best, to examine quintile-specific associations with our outcome. Because some variables tend to explain very similar constructs, composite indices have been created. “Percentage of individuals 150% below poverty level”, “percentage of family below poverty level”, and “median household income” have been grouped into a single index named “Poverty/Income”. “Percentage of foreign-born”, “percentage of language isolation” have also been grouped into a single index called “Foreign-born/language-isolation”. Additional county-level variables providing demographic characteristics were added to describe the metropolitan versus non-metropolitan nature of the county of residence as well as its region.

### 2.5. Statistical Analysis

The overall baseline characteristics were examined for significance using the chi-square test. Then, 5-year prostate cancer-specific survival was determined using the official software for the SEER database, SEER*Stat, and the log rank test was used to test for the difference in survival rate. The end of the follow-up status was either “dead” or “alive” and the intervals were described in months, totaling 60 months. Kaplan–Meier curves were plotted to examine 5-year survival for racial/ethnic groups in the lowest and highest SES quintiles and log-rank test to examine equality for survival functions. 

Cox-proportional hazard models assessed the relationships between county-level SES and PCa survival [31]. A multinomial logistic regression examining the relationship between SES and advanced disease (i.e., Distant stage and GS 8–10) and healthcare utilization (having a GS and undergoing PCa-directed surgery) was also performed. The model was adjusted for established and independent risk factors for poorer PCa survival, age, and marital status, and stratified by races/ethnicities to assess in-between group disparities. We also adjusted for county-level demographics as prescriber practices have been shown to vary across USA regions [32]. We used the variance inflation factor (VIF) to verify the absence of multicollinearity [33]. 

Because observations are located within the county of residence, geographical clustering was plausible and multilevel survival Cox proportional hazards models with mixed effects incorporating cluster-specific random effects that could potentially modify the baseline hazard function were performed, and an exponential distribution was specified. Because worse PCa can often be predicted by poorer access to care and more severe disease [12], individual and clinical-level factors measuring those were also included in a subsequent model. County-level SES were then introduced in the next and final model to examine the impact of their introduction on results from previous models.

Stata 16/IC [StataCorp] was used for statistical analyses. A two-tailed alpha = 0.01 was used to determine statistical significance. 

## 3. Results

Table 1 represents the baseline characteristics of SEER patients, stratified by race/ethnicity. The total number of participants was 239,613. Black populations expectedly exhibited the youngest age at diagnosis at 63.6 years. Five-year PCa survival ranged between 93% and 94% in all groups, except for AI/AN, which had the lowest survival (88%). AI/AN (7.77%) had the most advanced stage at diagnosis, followed by Black (5.13%), and Hispanic populations (5.03%), while APIs were found to have the highest proportion of GS ≥ 8. Although PCa-directed surgery was present in slightly less than half of the patients, Black populations and AI/AN were underwent one the least frequently. More than half of the groups had medical insurance at diagnosis, while AI/AN, Hispanic, and API populations had the highest percentage of Medicaid-insured patients. White populations recorded the lowest rates of uninsured patients, while Black populations recorded the highest (Table 1).

Hispanic patients resided in counties with the lowest educational attainment, with two-fifths (40.75%) reporting less than high school education (<HS) and only 13.05% reporting at least a bachelor’s-level education (≥BL). More than one-third of AI/AN and Black patients resided in counties with the highest poverty/income. Four-ninths of API (44.67%) and two-fifths of Hispanic (41%) patients resided in counties with the highest concentrations of foreign-born/language-isolated populations. Most of this population resided in Metropolitan counties. Around half of the White patients resided in the Western region, while around half of the Black patients resided in the South. The vast majority of Hispanic, API, and AI/AN patients resided in the West (Table 1). 

**Figure 1:**
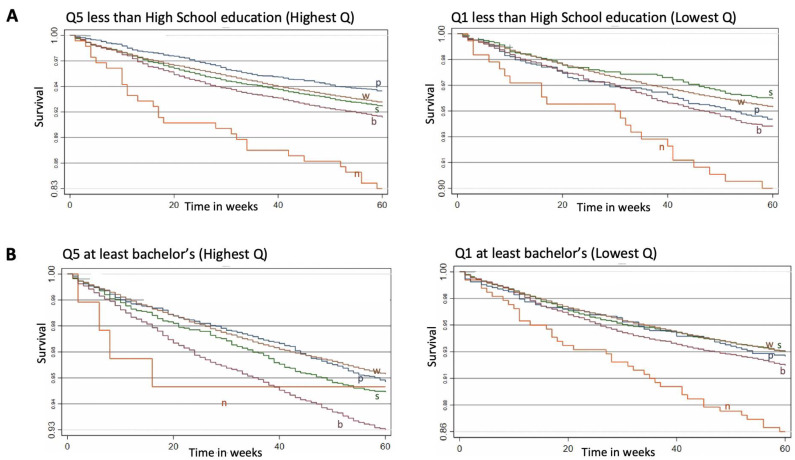

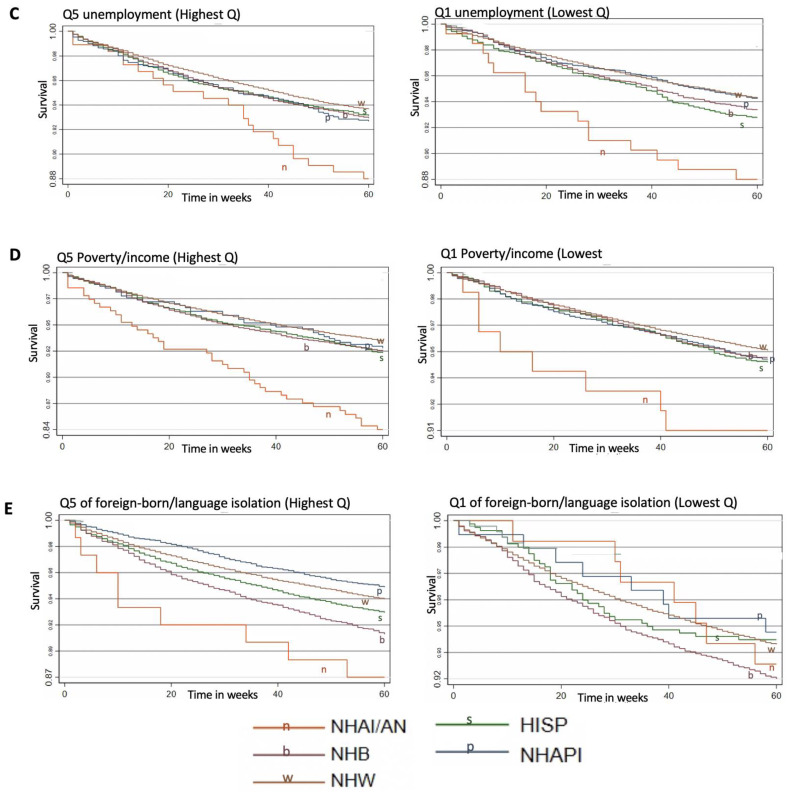
Cumulative survival of PCa from diagnosis stratified by racial/ethnic groups for each county-level SES, Q5 vs. Q1 for: less than high-school level education, (**A**), at least Bachelor-level (**B**), unemployment (**C**), poverty/income (**D**) and foreign-born/language isolated (**E**). Follow-up in all figures is 60 months = 5 years, *x* axis is time in weeks. Abbreviations: HISP = Hispanic population; NHAPI = Non-Hispanic Asian Pacific Islander population; NHAI/AN = Non-Hispanic American Indian/Alaska Native population; NHB = Non-Hispanic Black population; NHW = Non-Hispanic White population; Q = Quintiles. Scale of the graph is adjusted according to the survival values to allow easier visual detection.

Figure 1 provided Kaplan–Meier survival curves for individuals in the lowest and highest quintiles of SES variables stratified by racial/ethnic groups throughout the five-year follow-up. The log-rank test was statistically significant for all of the plots included. For each graph, observations were restricted to the most and the least deprived quintiles to illustrate racial/ethnic comparisons in survival throughout extreme SES variations. The lowest cumulative survival was observed within groups with the highest poverty rates and among those with the lowest educational attainment. AI/AN exhibited the poorest survival rates within all five of the most deprived SES quintiles. White patients had the highest survival even in counties with the worst unemployment and poverty/income rates. API populations had the highest survival in counties with the highest foreign/born-language isolation rates, while Hispanic patients had the highest survival in counties with the highest education attainment (Q1 of <HS) (Figure 1).

Table 2 represents the association between quintile-specific county-level SES and PCa-specific mortality, as well as variables illustrating disease severity and healthcare access/utilization, stratified by racial/ethnic categories. Overall, lower education, higher unemployment, and increased rates of poverty were associated with a higher risk of PCa mortality. For example, the poorest quintile of poverty/income increased PCa mortality by almost 40%. The protective association between higher education and reduced mortality remained for White, API, and Hispanic patients. Additionally, increased poverty rates were associated with poorer survival in patients reporting White, Black, and API race/ethnicity and living in the lowest poverty quintile increased the risk of PCa mortality by 74% among APIs. No within-group associations were detected between foreign-born/language isolation patients and PCa mortality, and no associations were found predicting PCa survival among patients reporting AI/AN race/ethnicity. 

Table 2 also examines the relationship between specific SES quintiles and disease severity (“Stage” and “GS”) and healthcare access/utilization (“GS not documented” and “surgery”). Overall, a higher risk of distant stage (vs. localized) was mainly associated with all included SES except for bachelor’s level education, while a higher risk of Gleason score > 7 was associated with poorer education and higher rates of unemployment. The associations between education and disease severity remained in patients reporting White or Hispanic race/ethnicity. Overall, living in Q5 of foreign-born/language-isolated populations was associated with an increased risk of advanced disease. Such association remained in White and Black populations, where the latter was at a 31% higher risk of having a distant-stage PCa at diagnosis (*p* < 0.01). 

Examining healthcare utilization, the performance of surgical intervention was associated with all socioeconomic factors. Similar associations between education and surgery were observed within racial categories and associations were the strongest when comparing extreme quintiles. For instance, AI/AN populations were 60% less likely to undergo surgery when residing in the highest educational quintile. Higher poverty rates were associated with an increased risk of surgical intervention. Patients reporting AI/AN race/ethnicity were almost twice as likely to have an undocumented GS when residing in either third, fourth, or fifth quintiles of poverty/income (*p* < 0.01). Higher education was associated with a reduced risk of having an undocumented Gleason Score. Adjusting for insurance did not significantly change the results obtained in Table 2.

Table 3 illustrates our multilevel analysis, which accounts for county-level clustering effects. Model 1 represents group-level Cox proportional HR, adjusted for marital status and age. Model 2 is adjusted for model 1 covariates and individual as well as clinical covariates. Model 3 is adjusted for model 2 covariates and county-level characteristics. We found racial/ethnic disparities across all racial/ethnic categories. 

Black populations (vs. White populations) were 1.6 times more likely to die from PCa in Model 1; however, such a disparity decreased to 1.19 (99%CI 1.12–1.27, *p* < 0.001) in Model 3. Hispanic men were at a higher risk of PCa mortality in model 1; however, upon adjusting for county-level and severity covariates, Hispanic populations exhibited a lower mortality than White population in subsequent models (0.90, 99%CI 0.83–0.97, *p* < 0.001). API populations were always at a lower mortality risk and their PCa mortality kept decreasing moving from Model 1 (0.79, 99%CI 0.70–0.88, *p* < 0.001) to Model 3 (0.67, 99%CI 0.69–0.75, *p* < 0.001). AI/AN populations had almost twice the mortality of White populations in Model 1, while this disparity was attenuated in subsequent models.

Intriguingly, racial/ethnic results were similar after adjusting for socioeconomic status (from Model 2 to Model 3) despite identifying evidence of protective effects of a university degree, increased risk due to being language-isolated, and reduced risk of death in those living in the Northeastern USA. In the final model and within county-level characteristics, only BL education (0.82, 99%CI 0.70–0.95, *p* = 0.001) and residing in the Northeast region (0.89, 99%CI 0.82–0.97, *p* < 0.001) were significantly associated and protective against PCa mortality. 

## 4. Discussion

The goal of the present study was to describe the degree to which county-level SES explained PCa survival in USA residents across a wide group of races and ethnicities. To our knowledge, this is the first study to examine the association between county-level SES and PCa-specific survival across five different racial/ethnic categories. In addition to other studies in the literature, our study included five racial/ethnic categories and examined how survival changes specifically in response to county-level SES profile in each racial/ethnic category included. Additionally, this study examined the potential pathway leading to worse PCa survival by demonstrating the influence of late-stage diagnosis and poorer healthcare utilization. Such an analysis highlights how PCa survival behaves in the function of race/ethnicity under different SES profiles and informs public health policies on potential county-level interventions to decrease the disparity gaps in PCa. Nonetheless, our results may have been conservative due to the standard and relatively shorter-term survival specified (5-year survival). Hence, larger effects are even expected when longer follow-up periods are involved.

Overall, lower area-level SES was associated with reduced PCa survival, which agreed with the literature examining a similar relationship, however, on a neighborhood level [20,21,22]. For example, a systematic review of 169 international publications established that men living in disadvantaged and/or rural areas face a greater PCa burden [34]. They also had consistently lower prostate-specific antigen (PSA) testing and PCa incidence, poorer survival, more advanced disease, and higher mortality [34]. DeRouen et al. also established a relationship between poorer neighborhood SES and poor PCa survival [21]. Our positive associations between county-level education and PCa survival were also similarly found, however, at the neighborhood level [22]. When fully adjusting our model for individual, clinical, and county-level SES (model 3, Table 3), PCa survival disparities between groups attenuated but remained, suggesting other factors may have contributed to survival disparities between groups. Those could have been related to behavioral factors and/or additional contextual factors such as rural/urban status, commuting and traffic patterns, residential mobility, and/or food environment [21,22].

Racially stratified findings in Table 2 illustrated within-group disparities in 5-year PCa-specific survival, suggesting varying county-level SES associations across race/ethnicity. White populations were more sensitive to changes in county-level SES, while the remaining groups exhibited less intense sensitivities. For example, and in contrast to White populations, better education did not influence PCa survival in Black populations, suggesting that interventions on such level might not turn out as beneficial for this minority group. Such findings could also support the diminishing returns hypothesis where minorities might not achieve the same health gains at higher SES as those of their White counterparts with similar SES [35,36,37]. For example, Kish et al. identified that mortality risk for Black patients increased with higher neighborhood SES when compared with White patients in the same SES quintile [20]. Furthermore, higher education was also found to be protective for advanced PCa among men residing in low SES, but not for men residing in neighborhoods within California with relatively higher SES [22], potentially suggesting the presence of a varying effect on PCa survival even under similar area-level characteristics. 

Nevertheless, education turned out to be an important significant factor in our population of Hispanic populations, as either HS-level or BL-level education, or even both were found to be significantly associated with PCa survival, disease severity (stage and GS), and/or healthcare utilization (surgery, GS documentation) (Table 2). This further demonstrates the variability in ethnic/racial response under similar SES circumstances, as Hispanic populations turned out to be mostly sensitive to education, which was not necessarily the case for their other racial/ethnic counterparts.

Analyses examining the association between SES and severity (stage and grade) as well as HC utilization (“GS not documented” and “surgery”) may provide partial early support for the view that these factors could play an intermediary role in the relationship between SES and PCa survival. Since worse stage/grade and lower HC utilization (surgery/GS documentation) were associated with poorer county-level SES in Table 2 and increased the risk of PCa mortality, the inclusion of those factors appears to strengthen the relationship between SES and PCa survival. More importantly, being insured was significantly protective against PCa mortality on multilevel analyses; however, adjusting for insurance did not significantly change the results between SES and disease severity and HC utilization. This might suggest that insurance is not necessarily affecting PCa survival via biopsy/surgery or that it instead matters more that patients have an earlier stage at diagnosis. Some studies suggested that while guaranteeing universal adequate health insurance is important, additional measures are needed to address persisting survival disparities [38,39]. Thus, interventions aiming at enhancing insurance status alone will likely not improve healthcare utilization for men residing in SES-deprived counties. It was also demonstrated that men residing in counties with the worse education rates were more likely to undergo PCa-directed surgery. Together, these findings could demonstrate how better education in PCa patients leads to more in-depth discussions with healthcare providers about additional less invasive options such as watchful waiting [40]. This was especially apparent in Hispanic populations where better education profiles were associated with better survival, lower disease severity, and lower likelihood of surgery and undocumented GS.

Finally, when adjusting for individual-level factors representing disease severity and HC access/utilization on multilevel analyses, disparities were diminished until they were even reversed in Hispanic populations (Model 2, Table 3). In fact, the magnitude of change was the highest when moving from Model 1 to Model 2 and within AI/AN and Black populations as the risk of PCa-specific mortality decreased from almost 1.88 to 1.35 and 1.64 to 1.22, respectively (Table 3). This could suggest that some minority groups might be more sensitive to individual-level factors as compared to others. The reversal of HR in Hispanic populations further demonstrates the “Hispanic Paradox”, where Hispanic populations continue to benefit from a life expectancy at least as high as Non-Hispanic White populations, even under lower SES [41,42,43]. Although speculative, given the lack of diet and exercise variables, the mortality advantage exhibited by Hispanic populations could be attributed to better dietary habits involving healthier food choices, and/or social and cultural support networks these groups maintain [44]. Hence, no single method of intervention would be closing all disparity gaps, as those should be carefully crafted and tailored based on each group’s needs. One could even argue that in some minority groups, individual-level factors could be driving poorer PCa survival in a way that better county-level SES cannot overcome. As an example, Du et al., found individual-level SES to be significantly accounting for disparities in PCa survival [45]. Furthermore, when adjusting for county-level characteristics, only BL education and the Northeast region were found to be significantly associated with PCa survival. This demonstrates the importance of BL education that remained apparent despite adjusting for more severe disease. Nevertheless, residing in the Northeast was protective, as this could be attributed to the fact that providers in the Northeast tend to have different prescribing practices than other regions and may be more likely to prescribe diagnostic and screening procedures, putting patients at a better likelihood for survival [32].

On the other hand, the protective effect of year could be explained by progress in advances in PCa management or could further back up recommendations against routine screening [46] since no increase in mortality was observed. It was surprising that unemployment exhibited non-significant associations with PCa survival in our final model. Since employment is tied with health insurance in the USA, we would normally expect lower HC access (and, hence, lower survival) caused by higher unemployment rates. However, PCa patients have a mean age between 63 and 64, and most of those patients would be retired. Thus, the detrimental effects of unemployment might not be as pronounced in this population of elderly patients. Still, this variable was included to test whether unemployment on a county level would show any effect on individual-level survival. 

API men appear to consistently have improved cancer survival rates as compared to White patients or to patients from other minority groups, while AI/AN men were shown to suffer from the worst PCa survival disparities. These results might emerge due to unobserved differences between groups, potentially due to either differences in the strength of social networks [47,48], or types of physical activity. Conversely, there could be genetic differences that explain variations in levels of risk between groups. Alternatively, White and Black patients residing in counties with the highest proportions of foreign-born/language-isolated might be at a higher risk for advanced-stage disease. Such a high proportion of foreign/born language-isolated may be likely to occupy more deprived areas [49], potentially contributing to the observed poorer PCa stage.

The poorest survival exhibited by AI/AN populations illustrates a minority group that is relatively rarely discussed in the literature, which warrants further attention, and enhanced public health and clinical focus. Stratified analyses did not show much SES association within the AI/AN group, which could be attributed to the smallest sample size of this group in comparison with others. Still, AI/AN men constitute the lowest proportions within the country, which could make them face larger burdens for social acceptability, health access and equity [50].

### Strengths and Limitations

This study has several strengths, including its population-based design that covers almost one-third of the American population and its linkage to SES characteristics. To our knowledge, this study is the first study to relate county-level SES with PCa outcomes including survival. We used multilevel Cox proportional hazards regression to account for geographic clustering of observations. However, although some suggest that census tract-level analyses might detect more accurate relationships between area-level factors and individual health outcomes [23,25], our adoption of the county-level might have been beneficial for delving deeper into disparities pathways. For example, Meliker et al. observed disappearing survival disparities in PCa between Black and White patients when moving their spatial analysis from larger scales (Federal/State Legislative Districts) to neighborhoods [51], potentially suggesting that smaller scales mimic “SES adjustment” in the USA. 

Despite several important strengths, this study has several limitations. Although missing data were kept at minimum in almost all covariates included, there was a large proportion of “unknown” values in Gleason score records. Despite this large percentage, the proportion with “unknown” score was stable same across racial/ethnic groups, thus perhaps suggesting that missing results are not related to one group or another. Nonetheless, the absence of time-varying covariates could have also affected the estimate as, for instance, some patients could have migrated to another geographical location just after diagnosis. Furthermore, the absence of chemotherapy/radiation therapy data represents another limitation. Nevertheless, access to those would have been also linked to insurance status, a variable that was accounted for. Additionally, the inclusion of other biopsy information, such as perineural invasion, would have been useful in assessing the relationship between multiple tumor aggressiveness indicators and PCa survival and variability across racial/ethnic groups studied. Nevertheless, GS and stage at diagnosis, included in the study, provide an objective assessment of severity of the disease. Another limitation of this study is that we examined gross treatment categories including the use of all-type surgical interventions. The specific subtype of surgical intervention may differ substantially in high-resourced hospitals and since different technologies may have different efficacies and impacts on the patient’s survival and wellbeing, thus future research should seek to determine whether treatment options differ between individuals with varying socioeconomic status. Lastly, the smaller sample of AI/AN as compared to the remaining groups could have impacted group comparability. Due to the small AI/AN population, a longer period of follow-up period (15 to 20 years) could be adopted in future research to better represent variability within that group.

## 5. Conclusions

Overall, this study provides insight into the impact of county-level SES on 5-year PCa survival as a risk of PCa-specific mortality for five ethnic/racial groups in the USA. Using the findings of this study could potentially inform the focus of public health efforts. As such, this study provides insight into the rising need to tailor interventions based on race/ethnicity and SES so that the benefit can be provided equitably. Future studies could benefit from performing mediation analysis for factors influencing the relationship between SES and PCa outcomes in order to more deeply understand the pathways leading to PCa disparities. Lastly, lengthening the follow-up period to around 15 to 20 years could reveal wider disparity gaps in outcomes when comparing the different racial/ethnic groups.

## Figures and Tables

**Table 1:** Baseline characteristics and 5-year survival of prostate cancer patients stratified into racial/ethnic groups.

	Overall (239,613)	Non-Hispanic White Populations (167,076)	Non-Hispanic Black Populations (34,654)	Hispanic Populations (20,865)	Non-Hispanic Asian Pacific Islander Populations (10,987)	Non-Hispanic American Indian/Alaska Native Populations (798)	*p*-Value
Mean Age at Diagnosis ± SD	65.40 ± 9.4	66.4 ± 9.4	63.6 ± 9.4	66.1 ± 9.6	68.1 ± 9.3	66.3 ± 9.2	<0.001
5-Year PCa Survival	0.94	0.94	0.93	0.93	0.94	0.88	<0.001
Year Of Diagnosis							
2007 (%)	50,641 (21.13)	36,084 (21.60)	6978 (20.14)	4188 (20.07)	2334 (21.24)	148 (18.55)	<0.001
2008 (%)	47,879 (19.98)	33,581 (20.10)	6884 (19.86)	4230 (20.27)	2145 (19.52)	158 (19.80)	
2009 (%)	48,485 (20.23)	33,491 (20.05)	7130 (20.57)	4445 (21.30)	2225 (20.25)	160 (20.05)	
2010 (%)	46,690 (19.49)	32,152 (19.24)	6876 (19.84)	4098 (19.64)	2150 (19.57)	174 (21.80)	
2011 (%)	45,918 (19.16)	31,768 (19.01)	6786 (19.58)	3904 (18.71)	2133 (19.41)	158 (19.80)	
Marital Status							
Divorced/Separated (%)	17,243 (7.20)	11,007 (6.59)	4089 (11.8)	1472 (7.06)	396 (3.6)	110 (13.79)	<0.001
Married (%)	157,828 (65.87)	115,628 (69.21)	18,481 (53.33)	13,425 (64.34)	8152 (74.20)	446 (55.89)	
Single (%)	22,925 (9.57)	13,574 (8.12)	6313 (18.21)	1973 (9.45)	694 (6.32)	69 (8.65)	
Widowed (%)	9286 (3.88)	6429 (3.85)	1512 (4.36)	846 (4.01)	406 (3.70)	35 (4.39)	
Unknown/Missing (%)	32,331 (13.49)	20,438 (12.23)	4259 (12.29)	3159 (15.14)	1339 (12.19)	138 (17.29)	
Insurance Status							
Any Medicaid (%)	10,204 (4.26)	3637 (2.18)	2504 (7.23)	2510 (12.03)	1300 (11.83)	121 (15.16)	<0.001
Insured (%)	202,120 (84.35)	146,063 (87.42)	28151 (81.23)	15719 (75.34)	8663 (78.85)	602 (75.44)	
Uninsured (%)	3215 (1.34)	1562 (0.93)	963 (2.78)	511 (2.45)	139 (1.27)	12 (1.50)	
Unknown/Missing (%)	24,080 (10.06)	15814 (9.47)	3036 (8.76)	2125 (10.18)	885 (8.05)	63 (7.89)	
Stage							
Localized (%)	191,882 (80.08)	191,882 (80.08)	28,263 (81.56)	15,887 (76.14)	8477 (77.15)	598 (74.94)	<0.001
Regional (%)	29,868 (12.46)	29,868 (12.47)	3675 (10.60)	2690 (12.89)	1544 (14.05)	95 (11.90)	
Distant (%)	9554 (3.99)	9554 (3.99)	1778 (5.13)	1049 (5.03)	508 (4.62)	62 (7.77)	
Unknown/Unstaged (%)	8309 (3.47)	8309 (3.47)	938 (2.71)	1239 (5.94)	458 (4.17)	43 (5.39)	
Gleason Score							
GS 6 (%)	56,158 (23.44)	40,209 (24.07)	7441 (20.9)	4929 (23.62)	2252 (20.50)	156 (19.55)	<0.001
GS 7 (%)	52,076 (21.73)	36,802 (22.03)	7852 (22.66)	4187 (20.07)	2391 (21.76)	153 (19.17)	
GS 8 (%)	9600 (4.01)	6352 (3.80)	1552 (4.48)	919 (4.40)	622 (5.66)	39 (4.89)	
GS 9 (%)	7516 (3.14)	5141 (3.08)	1104 (3.19)	676 (3.24)	504 (4.59)	27 (3.38)	
GS 10 (%)	913 (0.38)	623 (0.37)	134 (0.39)	112 (0.49)	49 (0.45)	1 (0.13)	
Not Done (%)	1126 (0.47)	726 (0.43)	175 (0.5)	132 (0.63)	70 (0.64)	10 (1.25)	
Unknown (%)	113,350 (47.30)	77,223 (46.22)	16,396 (47.31)	9920 (47.54)	5099 (46.41)	412 (51.63)	
Surgery							
Yes (%)	100,558 (41.97)	74,054 (44.32)	12,385 (35.74)	8636 (41.39)	4409 (40.13)	280 (35.09)	<0.001
No (%)	136,794 (57.09)	91,512 (54.77)	22,034 (63.58)	12,094 (57.96)	6425 (58.48)	515 (64.54)	
Unknown (%)	2267 (0.95)	1510 (0.90)	235 (0.68)	135 (0.65)	153 (1.39)	3 (0.38)	
Less Than High School Education							
Q1 (%) (Lowest “Less Than High School” Quintile)	49,326 (20.59)	41,067 (24.59)	4379 (12.64)	1339 (6.42)	1443 (13.14)	214 (26.82)	<0.001
Q2 (%)	48,547 (20.26)	37,373 (22.38)	5272 (15.21)	2247 (10.78)	2734 (24.89)	110 (13.78)	
Q3 (%)	48,134 (20.09)	31,903 (19.10)	7680 (22.16)	4333 (20.78)	3098 (28.20)	165 (20.68)	
Q4 (%)	47,575 (19.85)	30,224 (18.10)	10,544 (30.43)	4434 (21.27)	1178 (10.72)	140 (17.54)	
Q5 (%) (Highest “Less Than High School” Quintile)	45,944 (19.17)	26,451 (15.84)	6777 (19.56)	8495 (40.75)	2531 (23.04)	169 (21.18)	
At Least Bachelor-Level Education							
Q1 (%) (Lowest “At Least Bachelor-level” Quintile)	52,040 (21.72)	37,318 (22.34)	7785 (22.47)	5025 (24.10)	786 (7.16)	275 (34.46)	<0.001
Q2 (%)	43,561 (18.18)	31,416 (18.81)	8012 (23.12)	2152 (10.32)	1154 (10.51)	230 (28.82)	
Q3 (%)	47,261 (19.72)	29,430 (17.62)	5483 (15.82)	6625 (31.78)	3996 (36.38)	124 (15.54)	
Q4 (%)	49,600 (20.70)	35,640 (21.34)	6313 (18.22)	4326 (20.75)	1934 (17.61)	92 (11.53)	
Q5 (%) (Highest “At Least Bachelor-level” Quintile)	47,064 (19.64)	33,214 (19.89)	7059 (20.37)	2720 (13.05)	3114 (28.35)	77 (9.65)	
Unemployment							
Q1 (%) (Lowest “Unemployment” Quintile)	49,084 (20.48)	39,367 (23.57)	3728 (10.76)	2181 (10.46)	2907 (26.47)	137 (17.17)	<0.001
Q2 (%)	50,958 (21.27)	38,200 (22.87)	4953 (14.29)	3754 (18.01)	2683 (24.43)	216 (27.07)	
Q3 (%)	46,533 (19.42)	34,281 (20.53)	6225 (17.96)	3193 (15.32)	1874 (17.06)	110 (13.78)	
Q4 (%)	47,391 (19.78)	27,132 (16.24)	9461 (27.3)	6767 (32.46)	2445 (22.26)	157 (19.67)	
Q5 (%) (Highest “Unemployment” Quintile)	45,560 (19.01)	28,038 (16.79)	10,285 (29.68)	4953 (23.76)	1075 (9.79)	178 (22.31)	
Foreign-Born/Language-Isolated							
Q1 (%) (Lowest “Foreign-Born/Language-Isolated” Quintile)	48,022 (20.04)	37666 (22.55)	9148 (26.40)	607 (2.91)	144 (1.31)	98 (12.28)	<0.001
Q2 (%)	49,454 (20.64)	38637 (23.13)	7329 (21.15)	1672 (8.02)	708 (6.45)	291 (36.47)	
Q3 (%)	48,130 (20.09)	33885 (20.29)	7332 (21.16)	2856 (13.70)	3099 (28.21)	204 (25.56)	
Q4 (%)	47,392 (19.78)	31473 (18.84)	5066 (14.62)	7166 (34.37)	2126 (19.36)	134 (16.79)	
Q5 (%) (Highest “Foreign-Born/Language-Isolated” Quintile)	46,528 (19.42)	25357 (15.18)	5777 (16.67)	8547 (41.00)	4907 (44.67)	71 (8.90)	
Poverty/Income							
Q1 (%) (Lowest “Poverty/Income” Quintile)	48,402 (20.20)	36551 (21.88)	3508 (10.12)	3147 (15.09)	4110 (37.42)	79 (9.90)	<0.001
Q2 (%)	48,853 (20.39)	36349 (21.76)	5554 (16.03)	2924 (14.03)	2514 (22.89)	206 (25.81)	
Q3 (%)	46,533 (19.42)	35697 (21.37)	4541 (13.10)	3693 (17.71)	1461 (13.30)	153 (19.17)	
Q4 (%)	48,737 (20.34)	27589 (16.52)	9124 (26.33)	7980 (38.28)	2500 (22.76)	98 (12.28)	
Q5 (%) (Highest “Poverty/Income” Quintile)	47,001 (19.61)	30832 (18.47)	11925 (34.42)	3104 (14.80)	399 (3.63)	262 (32.84)	
County Description							
Metropolitan (%)	208,764 (89.14)	145,609 (87.18)	32,045 (93.52)	19,961 (95.75)	10,631 (96.79)	518 (73.58)	<0.001
Non-Metropolitan (%)	25,442 (15.86)	21,409 (12.82)	2607 (6.48)	887 (4.25)	353 (3.21)	186 (26.42)	
County Region							
Midwest (%)	23,057 (9.84)	18,348 (10.98)	4224 (12.19)	269 (1.29)	186 (1.69)	30 (3.76)	<0.001
Northeast (%)	41,093 (17.53)	31, 384 (18.78)	5657 (16.32)	3095 (14.83)	922 (8.39)	35 (4.39)	
South (%)	53,301 (22.74)	36,999 (22.15)	15,139 (43.69)	818 (3.92)	303 (2.76)	42 (5.26)	
West (%)	116,929 (49.89)	80,345 (48.09)	9634 (27.80)	16,683 (79.96)	9576 (87.16)	691 (86.59)	

Abbreviations: GS = Gleason Score; PCa = Prostate Cancer; Q = Quintile. The “Unknown” racial group was not represented in Table 1.

**Table 2:** Multivariable-adjusted hazards ratios examining risk of prostate cancer outcomes including cancer-specific mortality, disease severity and healthcare access and utilization via markers of socioeconomic status across the entire population and among racial/ethnic subgroups.

	Mortality			Distant Stage			Gleason Scores > 7			Received Surgical Intervention			Undocumented Gleason Score							
Versus Q1	Q2	Q3	Q4	Q5	Q2	Q3	Q4	Q5	Q2	Q3	Q4	Q5	Q2	Q3	Q4	Q5	Q2	Q3	Q4	Q5
Overall																				
Less than High School education	**1.10**	**1.23**	**1.27**	**1.33**	1.01	**1.19**	**1.13**	**1.29**	1.04	**1.11**	**1.10**	1.07	**1.07**	**0.83**	**1.06**	1.03	1.05	0.98	1.01	1.01
At least Bachelors-level education	**0.86**	**0.90**	**0.87**	**0.78**	0.95	1.06	0.98	0.92	1.01	0.99	1.03	**0.92**	0.98	**1.30**	**1.06**	**0.83**	0.98	0.97	**1.04**	0.97
Unemployment	0.98	**1.08**	**1.12**	**1.15**	0.97	1.05	**1.11**	1.07	1.03	1.06	1.01	**1.08**	**0.94**	**0.81**	**0.88**	**0.80**	1.03	0.99	0.99	1.02
Poverty/income	**1.17**	**1.23**	**1.31**	**1.38**	**1.13**	1.09	**1.24**	**1.19**	1.03	1.04	0.99	1.07	**1.05**	**1.16**	**1.11**	0.98	0.99	0.97	**0.92**	0.98
Foreign-born/Language-isolated	**0.90**	0.94	0.98	0.99	0.95	1.06	1.10	**1.21**	0.97	0.97	1.01	1.02	**1.06**	**0.78**	**0.93**	**0.90**	**1.09**	**1.05**	**1.09**	1.03
Non-Hispanic White population																				
Less than High School education	**1.12**	**1.17**	**1.18**	**1.31**	0.98	**1.12**	1.00	1.11	1.00	1.04	1.05	0.99	**1.08**	**0.89**	**1.18**	**1.14**	1.03	0.96	0.99	1.01
At least Bachelors-level education	**0.85**	**0.90**	**0.88**	**0.75**	0.96	1.05	1.05	0.94	1.01	0.97	1.06	**0.90**	0.98	**1.35**	1.04	**0.83**	0.99	0.99	**1.05**	0.98
Unemployment	0.96	1.04	0.98	1.06	0.94	0.97	0.90	0.94	1.04	1.07	0.96	1.05	0.97	**0.84**	1.01	**0.87**	1.02	0.99	1.01	1.03
Poverty/income	**1.14**	**1.18**	**1.19**	**1.20**	1.10	1.05	1.04	0.98	1.04	1.06	0.96	1.04	**1.09**	**1.23**	**1.31**	**1.10**	1.00	0.98	**0.95**	1.00
Foreign-born/Language-isolated	0.96	0.98	1.07	1.05	0.99	1.11	**1.15**	**1.21**	0.99	0.98	1.01	1.00	0.99	**0.75**	**0.89**	0.95	**1.08**	1.04	1.04	1.02
Non-Hispanic Black population																				
Less than High School education	0.98	1.15	1.17	1.23	0.97	1.11	1.14	1.22	1.06	1.13	1.09	1.20	**1.40**	1.05	**1.29**	**1.47**	1.09	1.01	1.02	0.99
At least Bachelors-level education	0.88	1.05	0.86	0.95	1.08	1.22	0.93	1.11	1.03	1.07	1.00	0.89	1.05	**1.26**	0.98	**0.75**	0.92	0.96	1.03	**0.89**
Unemployment	1.08	1.16	1.20	1.16	1.12	1.25	1.25	1.22	0.94	1.03	1.00	1.03	1.07	**0.82**	0.91	**0.86**	1.04	1.01	0.96	0.96
Poverty/income	**1.27**	1.24	**1.37**	**1.34**	1.18	1.09	**1.33**	1.28	1.06	1.06	1.05	1.10	1.01	1.06	1.05	**1.13**	1.09	1.05	**0.86**	0.95
Foreign-born/Language-isolated	0.92	0.98	0.98	1.18	1.03	1.08	1.00	**1.31**	0.98	**0.84**	0.90	0.95	**1.14**	**0.68**	0.98	0.94	1.10	0.97	**1.13**	0.94
Hispanic population																				
Less than High School education	**1.52**	1.38	**1.55**	**1.51**	1.43	1.34	1.41	**1.58**	1.46	**1.59**	**1.62**	1.35	0.91	**0.79**	1.13	0.99	1.05	0.92	0.93	0.93
At least Bachelors-level education	0.89	**1.20**	1.10	0.88	0.72	1.19	1.21	0.83	0.90	0.93	1.03	1.04	**0.73**	1.11	0.91	**0.71**	1.11	**0.88**	0.96	1.06
Unemployment	0.84	0.92	1.05	0.91	1.02	1.31	1.29	1.09	1.22	1.20	1.12	1.21	0.97	**0.80**	0.91	0.94	1.03	0.94	0.91	0.98
Poverty/income	1.95	1.10	1.21	1.19	1.11	1.03	1.15	1.10	1.07	1.12	0.96	1.18	**1.23**	1.13	**1.21**	1.09	0.90	**0.87**	**0.84**	0.97
Foreign-born/Language-isolated	1.23	1.20	1.14	1.24	0.95	0.88	1.07	1.16	0.95	1.00	1.06	0.97	1.12	0.89	0.96	0.93	1.13	1.21	1.23	1.14
Non-Hispanic Asian Pacific Islander population																				
Less than High School education	0.88	1.03	1.29	1.11	0.74	0.77	0.79	0.98	0.82	0.83	0.81	0.80	1.15	0.84	**1.40**	**1.29**	1.20	1.13	1.22	1.06
At least Bachelors-level education	0.82	**0.61**	0.76	**0.59**	1.02	0.80	0.72	0.67	0.96	0.97	0.94	0.98	0.84	**1.47**	**1.30**	**0.78**	0.95	**0.80**	0.83	**0.81**
Unemployment	1.16	1.13	1.06	**1.74**	1.08	0.88	1.15	1.25	1.04	0.81	0.88	0.86	**0.76**	0.86	1.06	0.86	0.99	0.94	0.89	1.18
Poverty/income	1.23	**1.49**	1.16	**1.74**	1.09	1.15	1.19	1.48	0.96	0.85	0.95	**0.55**	1.00	1.07	**1.30**	0.87	0.98	1.03	0.91	1.25
Foreign-born/Language-isolated	0.76	0.95	1.24	0.95	0.74	1.08	1.18	0.99	0.79	0.93	0.99	0.90	0.90	0.94	0.88	0.83	1.00	0.96	0.96	0.88
Non-Hispanic American Indian/Alaska Native population																				
Less than High School education	0.65	1.57	1.11	1.68	0.29	0.64	1.24	1.12	1.80	1.55	1.28	1.03	0.59	0.58	0.77	0.77	1.23	1.05	1.63	1.57
At least Bachelors-level education	1.1	0.90	0.80	0.55	0.79	0.60	0.74	0.40	1.42	0.73	0.97	0.87	0.72	0.86	0.78	**0.40**	0.95	1.05	0.69	0.61
Unemployment	0.59	0.80	2.24	1.34	0.41	0.45	1.35	0.52	1.28	1.99	1.71	2.30	0.82	0.95	0.89	1.03	0.65	0.61	1.09	0.64
Poverty/income	0.78	1.11	1.28	1.43	1.09	0.72	0.75	2.01	0.77	1.70	0.39	1.22	1.04	1.16	0.89	1.13	1.37	**2.10**	**2.19**	**1.97**
Foreign-born/Language-isolated	1.49	2.00	1.49	2.20	1.96	1.82	1.32	1.43	1.61	0.83	1.39	0.97	0.67	0.49	0.68	0.44	1.21	1.21	1.13	1.35

GS = Gleason Score; PCa = Prostate Cancer; Q# = Quintile. All analyses adjusted for age, marital status, metropolitan vs. non-metropolitan counties and county region. In models assessing inequalities in surgery, we also adjusted for stage and Gleason Score. Adjusting for insurance status did not significantly change the results. Values with *p*-value < 0.01 are bolded.

**Table 3:** Multilevel Cox proportional hazards modeling incorporating random intercepts for unobserved county-level variability using the SEER.

	Model 1				Model 2				Model 3			
	aHR	*p*-Value	Upper 99%CI	Lower 99%CI	aHR	*p*-Value	Upper 99%CI	Lower 99%CI	aHR	*p*-Value	Upper 99%CI	Lower 99%CI
Race/ethnicity (ref = Non-Hispanic White population)												
Non-Hispanic Black population	1.64	<0.001	1.54	1.74	1.22	<0.001	1.14	1.30	1.19	<0.001	1.12	1.27
Hispanics	1.11	<0.001	1.03	1.20	0.89	<0.001	0.83	0.96	0.90	<0.001	0.83	0.97
Non-Hispanic Asian Pacific Islander population	0.79	<0.001	0.70	0.88	0.66	<0.001	0.59	0.74	0.67	<0.001	0.60	0.75
Non-Hispanic American Indian/Alaska Native population	1.88	<0.001	1.42	2.48	1.35	0.006	1.02	1.79	1.33	0.007	0.99	1.76
Individual-level characteristics												
Stage at diagnosis (ref = local)												
Regional					2.74	<0.001	2.50	2.98	2.74	<0.001	2.51	2.99
Distant					24.78	<0.001	23.44	26.18	24.74	<0.001	23.42	26.14
Gleason Score (ref = GS6)												
GS7					1.96	<0.001	1.71	2.25	1.96	<0.001	1.71	2.24
GS8					4.27	<0.001	3.73	4.91	4.27	<0.001	3.73	4.91
GS9					6.92	<0.001	6.07	7.86	6.90	<0.001	6.06	7.86
GS10					10.56	<0.001	8.94	12.48	10.47	<0.001	8.86	12.36
Not carried out/unknown					6.89	<0.001	4.11	5.27	6.89	<0.001	4.11	5.27
Surgery (ref = no)												
Yes					0.54	<0.001	0.50	0.58	0.54	<0.001	0.50	0.58
Year of diagnosis												
2011 vs. 2007					0.91	<0.001	0.90	0.93	0.91	<0.001	0.90	0.93
Insurance status at diagnosis (vs. no insurance)												
Any Medicaid					0.68	<0.001	0.59	0.79	0.68	<0.001	0.59	0.79
Insured					0.44	<0.001	0.38	0.50	0.44	<0.001	0.39	0.50
County-level characteristics												
County-level SES (Q5 vs. Q1)												
Less than high school									0.97	0.633	0.88	1.15
At least bachelor-level									0.82	0.001	0.70	0.95
Unemployment									0.96	0.273	0.87	1.06
Poverty/Income									1.10	0.091	0.95	1.27
Foreign-born/Language-isolated									1.14	0.043	0.96	1.36
County metro/non-metro												
Non-metropolitan (vs. metro)									1.03	0.459	0.94	1.16
County region (vs. West)												
Midwest									1.00	0.982	0.89	1.13
Northeast									0.89	<0.001	0.82	0.97
South									1.10	0.013	0.97	1.21

Model 1: adjusted for marital status and age; Model 2: adjusted for model 1 covariates and clinical and individual-level characteristics; Model 3: adjusted for model 2 covariates and county-level socioeconomic and demographic characteristics; abbreviations: GS = Gleason Score, PCa = Prostate Cancer, Q = Quintile.

## Data Availability

The SEER database is publicly available, with free access, in case additional information is needed regarding data analysis. We can share the STATA code upon receipt of any request in an email to the corresponding author.
